# Appearance of the bladder on initial voiding cystogram in boys with PUV and its relation to pre and postnatal findings

**DOI:** 10.3389/fped.2024.1380502

**Published:** 2024-04-18

**Authors:** S. Pecorelli, C. Ferdynus, J. Delmas, L. Harper

**Affiliations:** ^1^ Department of Pediatric Urology, Hôpital Pellegrin-Enfants, CHU Bordeaux, France; ^2^ Methodological Support Unit, Reunion University Hospital, La Réunion, France; ^3^ Clinical Informatics Department, Reunion University Hospital, La Réunion, France; ^4^ Clinical Research Department, INSERM CIC1410, La Réunion, France; ^5^ Department of Pediatric Radiology, Hôpital Pellegrin-Enfants, CHU Bordeaux, France

**Keywords:** posterior urethral valves, cystogram, bladder function, diagnosis, urodynamics

## Abstract

**Introduction:**

Bladder profile in boys with Posterior Urethral Valves can be very varied with a spectrum going from high pressure, unstable, hypocompliant small bladders to hypercompliant, large acontractile bladders, with some being near-normal. Our question was whether appearance, specifically of the bladder, on initial VCUG was correlated to prenatal features and whether it could predict early postnatal outcome.

**Method:**

We used a prospectively gathered database of boys with prenatally suspected PUV. We analyzed whether the appearance, specifically of the bladder, was related to date of prenatal diagnosis, presence of a megacystis on prenatal ultrasound, presence of vesico-ureteral reflux (VUR), presence of abnormal DMSA scan, nadir creatinine or presence of febrile urinary tract infection (fUTI) during the first two years of life.

**Results:**

The database comprised 90 cystograms. 15% of bladders were judged normal/regular, 54 % were small/diverticular and 31% were large/diverticular. Bladder appearance was not associated with presence of prenatal megacystis, abnormal DMSA scan, VUR, nor rate of fUTI. The only significant associations were normal/regular bladder and early prenatal diagnosis (*p* = 0.04) and normal/regular bladder and elevated nadir creatinine (>75µmol/l) (*p* = 0.01).

**Discussion:**

We believe that when focusing solely on the appearance of the bladder, excluding information about the urethra and presence of reflux, the cystogram alone is insufficient to inform on future bladder function. This could be used as an argument in favor of performing early urodynamics in this population.

## Introduction

Posterior urethral valves (PUV) represent the most common cause of Lower Urinary Tract Obstruction (LUTO) in boys. They affect 1:4,000–1:25,000 births, and cause increased intravesical pressure during fetal kidney development resulting in various degrees of kidney and bladder impairment (1–3). We know that bladder profile in boys with PUV can be very varied both in the newborn period, but also with time, with a spectrum going from high pressure, unstable, hypocompliant small bladders to hypercompliant, large acontractile bladders, with some being near-normal. We know that bladder behavior changes during childhood, particularly in boys with PUV, and some bladders will go from one to profile to another though this generally happens after a few years (4). What is less known is if the initial presentation of the bladder predicts early clinical outcome.

Early invasive urodynamics could give us detailed information about bladder function but these exams are not systematically performed in neonates. On the other hand, most if not all boys with suspected PUV undergo a diagnostic voiding cystourethrogram (VCUG). Our question was therefore whether the appearance of the bladder on initial VCUG was correlated to specific prenatal features and more importantly whether it could predict early postnatal outcome (within 2 years of age). The hypothesis was that an abnormal bladder appearance on cystogram would be more frequently associated with adverse outcomes or associated anomalies.

## Patients and method

We used a prospectively gathered database of boys with prenatally suspected, postnatally confirmed PUV. We analyzed whether the appearance of the bladder was related to date of prenatal diagnosis (before or after 28 weeks gestation) or presence of a megacystis on prenatal ultrasound. Megacystis was defined as a bladder diameter of: >7 mm in the 1st trimester, >30 mm in the 2nd trimester, >60 mm in the 3rd trimester (5). We also analyzed whether appearance of the bladder was associated with presence of vesico-ureteral reflux (VUR), or presence of abnormal DMSA scan. Finally, we looked at whether appearance of the bladder was correlated with nadir creatinine (lowest creatinine within the first year of life) or presence of febrile urinary tract infection (fUTI) during the first two years of life.

Using the radiologist reports, bladders were categorized as small/diverticular, normal/regular, or large/diverticular according to whether they were <70%, 70%–130% or >130% of estimated weight-adjusted bladder volume (calculated using the formula weight in kilograms × 7). Though the aspect of the urethra had obviously confirmed the diagnosis of PUV, we looked specifically at the appearance of the bladder. All cystograms were performed within the first week of life. Cystograms were performed following the recommendations of the European Society of Pediatric Radiology (6). All DMSA scans were performed within 3 months of life. The DMSA scan was considered abnormal if there was heterogeneity suggestive of cortical anomalies, or if there was a difference in differential function >10%. The nadir creatinine was the lowest creatinine within the first 2 years of life. Statistical analysis was performed using the chi-squared test for independence. Febrile urinary tract infection was defined as presence of fever (>38.5 °C) and positive urine culture.

All children underwent clinical, biological and radiological follow-up at 1, 3, 6, 12, 18 and 24 months. They all underwent a DMSA scan between 2 and 6 months of age and were all under antibiotic prophylaxis. Decisions on specific bladder treatment including anticholinergics and/or introduction of CIC were left to the surgeons managing the child.

## Results

Patients were included between 2012 and 2017. All children were identified prenatally and had postnatally confirmed PUV. Average gestational age at diagnosis was 28.6 (±5) weeks, average gestational age at birth was 37.9 (±1.7) weeks, average birth weight was 3.2 kg (±522 g). Valve resection took place at a mean 7 (±9) days post-birth. Follow-up was 2 years. The database comprised 90 cystograms. 15% of bladders were judged normal/regular, 54% were small/diverticular and 31% were large/diverticular. As there were 6 distinct outcomes, we noted in the table when there was missing data for each outcome (Table 1).

**Table 1:** Results.

	Missing data	Normal (*n* = 13)	Small/diverticular (*n* = 49)	Large/diverticular (*n* = 28)	*p*-value
Megacystis	15				
No		6/12 (50.0%)	30/40 (75.0%)	18/23 (78.4%)	0.17
Yes		6/12 (50.0%)	10/40 (25.0%)	5/23 (21.7%)	
Abnormal DMSA scan	21				
No		3/8 (37.5%)	15/41 (36.6%)	7/20 (35.0%)	0.99
Yes		5/8 (62.5%)	26/41 (59.4%)	13/20 (65.0%)	
VUR	4				
No		5/13 (38.5%)	20/47 (42.5%)	13/26 (50.0%)	0.75
Grade 3–5		8/13 (61.5%)	27/47 (57.5%)	13/26 (50.0%)	
Nadir Creat	0				
<75 µmol/L		9/13 (69.2%)	46/49 (93.9%)	27/28 (96.4%)	0.01
≥75 µmol/L		4/13 (30.8%)	3/49 (6.1%)	1/28 (3.6%)	
fUTI	0				
No		10/13 (76.9%)	43/49 (87.8%)	19/28 (67.9%)	0.11
Yes		3/13 (23.1%)	6/49 (12.2%)	9/28 (32.1%)	
Prenatal Diagnosis	11				
2nd trimester		9/12 (75.0%)	15/44 (34.1%)	9/23 (39.1%)	0.04
3rd trimester		3/12 (25.0%)	29/44 (65.9%)	14/23 (60.9%)	

Bladder appearance was not associated with presence of prenatal megacystis, abnormal DMSA scan, nor VUR, which was present in 55% of cases regardless of bladder type. Bladder type was not associated with rate of fUTI though presence of VUR was associated with fUTI. The only significant associations were normal/regular bladder and early prenatal diagnosis (*p* = 0.04) and normal/regular bladder and elevated nadir creatinine (>75 µmol/L) (*p* = 0.01). It was not however possible to distinguish whether the latter association was due to the relation between early diagnosis and nadir creatinine or whether this was a direct association.

## Discussion

In our series, bladder distribution favored small bladders and only 15% were judged normal. Though a normal looking bladder could be in theory reassuring, in our series it was associated with more severe forms. This could either be interpreted as meaning that the supposedly normal aspect at initial cystogram was just a transitory aspect in these bladders that were diagnosed early and have evolved, or just simply that bladder morphology on initial cystogram gives no reliable data.

Indeed, it must be said that a voiding cystogram is a morphological exam not a functional one. It gives no information on bladder wall thickness, compliance, or filling pressure. How the bladder and upper urinary tract adjusted to the increased pressure during bladder and upper urinary tract development is specific to each child. Some might have an enlarged bladder without VUR whilst other will have a normal sized bladder with VUR depending on how their urinary tract reacted. So appearing normal does not mean the function is normal. Furthermore, bladder evolves post-resection and during the first months and years and its appearance just after birth does not necessarily determine its future evolution (4). The only test currently available to investigate bladder function at any given time is urodynamic testing.

Secondly, there is certainly wide variation in how cystograms are performed. Although there are standardized protocoles (6, 7), the gravity bladder-filling pressure used can be variable, and voiding during the exam can influence interpretation of bladder volume. Presence or absence of the catheter also influences aspect and its filling. The aspect of the bladder can vary according to how the cystogram was performed. Recent studies have shown that even the actual diagnosis of PUV can be missed on a voiding cystogram. Unsuspicious findings of the urethra on VCUG cannot exclude abortive forms of PUV (8). Though the absence of any radiological signs of PUV on VCUG excludes PUV with a very high negative predictive value, considering indirect signs can improve detection rate (9).

Nevertheless, several teams have tried to gather information from the appearance of the bladder on cystogram. There have been several studies performed in children with neurogenic bladders, studying bladder height-to-width ratio in order to predict high-pressure bladders. The idea is that because of bladder muscle fiber anatomy, the bladder elongates with high-pressure or large volumes (10). However, in the study by Kumano et al. for instance, the specificity of height to width ratio was low at 59% and there was no significant difference in maximum bladder volume between high-pressure and low-pressure bladders. Babu et al. have also recently published a study on height to width ratio in boys with posterior urethral valves with interesting results, though as opposed to neurogenic bladders, the actual scope of bladder anomalies in PUV patients is wider (11). The study by Wu et al. looked at various parameters such as height to width ratio of the bladder (HW-B), height to width ratio of the posterior urethra (HW-PU), posterior-anterior urethral ratio (PA-UR), bladder trabeculation grade, and presence/laterality of vesicoureteral reflux on early cystograms, but found that only HW-PU was related to nadir creatinine (12). Other teams have proposed alternative classifications of the PUV bladder in an attempt to predict outcome. The report by Niyogi et al. describes a Shape, Wall, reflux and Diverticuli (SWRD) score and correlate it to function, but these were images taken during videourodynamics (VUD) in children aged between 1 and 14 years of age with a mean age of 6 (13). The cystograms used were not the original diagnostic cystograms but VUD images. Though they do find some correlation, they agree that the SWRD score cannot be used alone for decision-making. More recently, the study published by Archana et al. tried to identify surrogate-imaging markers of urodynamic proven bladder dysfunction in PUV (14). They determined a “bladder hostility score” based on bladder contour, grade of VUR, bladder neck appearance and posterior/anterior urethral ratio. Their population was also older with an age range of 3–17 years of age though they did use the diagnostic cystogram. Their conclusion was however, the same as ours as they state that imaging parameters are unable to convincingly identify the type of bladder dysfunction required to manage PUV patients appropriately, and that urodynamic testing remains the gold standard investigation to assess bladder dysfunction. The study published in 2016 by Hochart et al. questioning whether neonatal imaging findings were predictive of renal function during early childhood, and which studied both ultrasound and cystogram found that neither were predictive of renal failure at 3 years (15).

Our study has limitations. Bladders were categorized into three groups according to bladder appearance alone and that this can seem too simplistic and we did not use combined markers as done in some of the aforementioned studies. We chose to use this simple classification to be as close as possible to the way cystograms are analyzed in daily practice and because PUV bladders can adopt very different morphologies, some are small and some are large.

We did not perform systematic urodynamics in our population so we do not correlate aspect of the bladder with urodynamic findings but with clinical outcome and associated findings. However, we do believe that clinical outcome is main the driver of medical management. The strength of the study is the standardized care these children received, as they were included in a concomitant randomized trial, including a strict definition of febrile urinary tract infection, systematic DMSA scan and regular follow-up.

In conclusion, we believe that when focusing solely on the appearance of the bladder, excluding information about the urethra and presence of reflux, the cystogram alone is insufficient to inform on future bladder function. This could be used as an argument in favor of performing early urodynamics in this population.

## Data Availability

The raw data supporting the conclusions of this article will be made available by the authors, without undue reservation.
